# Association between Cu/Zn/Iron/Ca/Mg levels and cerebral palsy: a pooled-analysis

**DOI:** 10.1038/s41598-023-45697-w

**Published:** 2023-10-27

**Authors:** Haiquan Zhu, Song Mao, Wei Li

**Affiliations:** 1 Department of Orthopaedics, The Affiliated Lianyungang Hospital of Xuzhou Medical University, The First Affiliated Hospital of Kangda College of Nanjing Medical University, Lianyungang Clinical College of Nanjing Medical University, The First People’s Hospital of Lianyungang, Lianyungang, Jiangsu China; 2 Department of Pediatrics, Shanghai Sixth People’s Hospital, Affiliated to Shanghai Jiao Tong University School of Medicine, Shanghai, China; 3 Department of General Surgery, The Affiliated Lianyungang Hospital of Xuzhou Medical University, The First Affiliated Hospital of Kangda College of Nanjing Medical University, Lianyungang Clinical College of Nanjing Medical University, The First People’s Hospital of Lianyungang, Lianyungang, Jiangsu China

**Keywords:** Biomarkers, Health care

## Abstract

It was well documented that macro/trace elements were associated with the neurodevelopment. We aimed to investigate the relationship between copper (Cu)/zinc (Zn)/iron/calcium (Ca)/magnesium (Mg) levels and cerebral palsy (CP) by performing a meta-analysis. We searched the PubMed, Embase, Cochrane and Chinese WanFang databases from January 1985 to June 2022 to yield studies that met our predefined criteria. Standard mean differences (SMDs) of Cu/Zn/Iron/Ca/Mg levels between CP cases and healthy controls were calculated using the fixed-effects model or the random-effects model, in the presence of heterogeneity. 95% confidence intervals (CI) were also computed. Sensitivity analysis was performed by omitting each study in turn. A total of 19 studies were involved in our investigation. CP cases showed markedly lower Cu, Zn, iron and Ca levels than those in controls among overall populations (SMD =  − 2.156, 95% CI − 3.013 to − 1.299, *P* < 10^−4^; SMD =  − 2.223, 95% CI − 2.966 to − 1.480, *P* < 10^−4^; SMD =  − 1.092, 95% CI − 1.513 to − 0.672, *P* < 10^−4^; SMD =  − 0.757, 95% CI − 1.475 to − 0.040, *P* = 0.038) and Asians (SMD =  − 2.893, 95% CI − 3.977 to − 1.809, *P* < 10^−4^; SMD =  − 2.559, 95% CI − 3.436 to − 1.683, *P* < 10^−4^; SMD =  − 1.336, 95% CI − 1.807 to − 0.865, *P* < 10^−4^; SMD =  − 1.000, 95% CI − 1.950 to − 0.051, *P* = 0.039). CP cases showed markedly lower Zn level than that in controls among Caucasians (SMD =  − 0.462, 95% CI − 0.650 to − 0.274, *P* < 10^−4^). No significant differences of Cu, iron and Ca levels between CP cases and controls among Caucasians (SMD =  − 0.188, 95% CI − 0.412 to 0.037, *P* = 0.101; SMD =  − 0.004, 95% CI − 0.190 to 0.182, *P* = 0.968; SMD = 0.070, 95% CI − 0.116 to 0.257, *P* = 0.459) were observed. No marked difference of Mg level between CP cases and controls was noted among overall populations (SMD =  − 0.139, 95% CI − 0.504 to 0.226, *P* = 0.455), Asians (SMD =  − 0.131, 95% CI − 0.663 to 0.401, *P* = 0.629), and Caucasians (SMD =  − 0.074, 95% CI − 0.361 to 0.213, *P* = 0.614). Sensitivity analysis did not change the overall results significantly for Cu, Zn, iron and Mg. CP cases demonstrated significantly lower levels of Cu/Zn/iron/Ca than those in healthy controls, particularly in Asians. Decreasing trend of Cu/Zn/iron/Ca levels merit attention, particularly in the population with high susceptibility to CP. Frequent monitoring and early intervention may be needed.

## Introduction

Cerebral palsy (CP), a group of motor disorders and cognitive disturbances, is an important health concern in the newborns, particularly in the premature or low-birthweight neonates^1^. CP is likely to lead to the musculoskeletal problems, even epilepsy^2^. Many children with CP have limitations during their daily activities, including feeding, dressing, and balance. Some CP cases are complicated with malnutrition. CP Progression results in an increased morbidity and mortality, which indicates that early prevention and intervention for CP is of great importance. Strenuous efforts have been made to identify the risk factors for CP susceptibility. Birth asphyxia and genetic factors were involved in the development of CP^3^. In spite of the progress of the prenatal diagnosis and interventions, the prevalence of CP did not decline obviously, which indicated that CP may be a multi-factor disease, an in-depth investigation of other potential risk factors for CP is imperative.

Trace elements status is closely associated with the immune system function via their effects on many biological Processes, while the well worked immune function required the micronutrients participating in cell metabolism and replication. For instance, leukocytes proliferation induced by acute infection was impaired by insufficient supply of trace elements, including iron, zinc, magnesium and manganese^4^. Trace elements also exerted effects on the cellular transfer and the levels of other important nutrients^5^. For example, iron was an important constituent of hemoglobin, which carried the oxygen and participated in the energy metabolism. It is also proved that certain trace elements affect the chemical synaptic transmission in the brain and peripheral central nervous system^6^. Cu and Zn play an important role in the activation of enzymes that are involved in catecholamine transmission. On the other hand, macro-elements, such as Ca and Mg play an important role in the physical development. Ca and Mg exert effects in the transmission of neural stimuli.

Based on the fact that CP is essentially a neurological disorder, we speculated that macro/trace elements levels may be associated with CP. Some available evidence showed that certain trace elements, such as copper (Cu) deficiency were correlated with learning and behavior disorders^7^. Meanwhile, markedly lower level of zinc (Zn) was observed in severe CP compared with that in controls^8^. Previous review showed a high rate of malnutrition in the children with CP, while hypocalcemia, reduced serum levels of Zn, Cu and vitamin D being reported the most^9^. Mg sulfate given antenatally in threatened preterm labor has a reduction in the risk of CP at 2 years of age^10^. The administration of vitamin D and Ca produced a large, nonsignificant effect on bone mineral density in the lumbar spine^11^. To have an in-depth understanding of the relationship between alterations of macro/trace elements and CP is helpful for CP prevention and intervention.

Meta-analysis is a good way to pool the available evidence from single study to produce a more comparatively robust result, which increases the statistical power significantly. Therefore, we conducted a meta-analysis with the aim of clarifying the differences of Cu/Zn/Iron/Ca/Mg levels between CP and healthy controls in children.

## Materials and methods

### Search strategy

We performed the literature search in terms of the preferred reporting items for systematic reviews and meta-analysis guidelines^12^, we searched the papers that reported the levels of Cu/Zn/Iron/ Ca/Mg, both in CP and healthy controls from January 1985 to June 2022 by using PubMed, Embase, Cochrane and Chinese WanFang databases. We used the searched terms as follows: (1) macro/trace element, micronutrient, magnesium, Mg, calcium, Ca, iron, zinc, Zn, copper and Cu; (2) urine, serum and plasma; and (3) cerebral palsy, CP. We also reviewed the references of extracted literature. The paper with the larger number of participants was enrolled if the same subjects were recruited in more than one study. Our preprint of “Association between Cu/Zn/Iron/Ca/Mg levels and Chinese children with cerebral palsy” (https://doi.org/10.21203/rs.3.rs-703495/v1) was stored in websites (researchgate.net/publication/353611059_Association_between_CuZnIronCaMglevels_and_Chinese_children_with_cerebral_palsy), (doc.taixueshu.com/search?sourceTye = all&keywordTyPe = 1&keyword = Association + between + Cu/Zn/Iron/Ca/Mg + levels + and + cerebral + palsy&resultSearch = 0).We cited this preprint. This preprint has not been published in whole or in part in any formal journal elsewhere.

### Study selection criteria

1. Study design: case–control study
2. Case: cerebral Palsy, control: healthy participants
3. Outcome of interest: Cu/Zn/Iron/Ca/Mg levels in cases and controls

### Exclusion criteria

1. Study design: case report, comment, editorials and reviews
2. Case and control: lack of detailed number of cases and controls, multiple publications of the same data
3. Outcome of interest: lack of detailed data of Cu/Zn/Iron/Ca/Mg levels

### Data extraction

We collected the data of mean and standard deviation (SD) of Cu/Zn/Iron/Ca/Mg levels. We also extracted the study characteristics from enrolled investigations. Data were recorded as the followings: first author’s last name; year of publication; ethnicity; number of cases and controls; confounding factors and testing method of Cu/Zn/ Iron/Ca/Mg levels.

### Statistical analyses

We used the standard mean difference (SMD) to test the differences of Cu/Zn/ Iron/Ca/Mg levels between CP cases and controls across studies. Heterogeneity of SMDs across studies was tested by using the Q statistic (significance level at *P* < 0.05). The I^2^ statistic, a quantitative measure of inconsistency across studies, was also calculated. The SMDs were calculated using either fixed-effects models or, in the presence of heterogeneity, random-effects models (Q test, *P* < 0.05). Sensitivity analysis was performed by omitting each study in turn. Potential publication bias was assessed by Egger’s test at the *P* < 0.05 level of significance if the number of recruited studies were more than 10. Trim and fill analysis was used to identify the funnel plot asymmetry caused by publication bias and test the solidity of the results. All analyses were Performed using STATA version 12.0 (Stata Corp, College Station, TX).

## Results

### Literature search

**Figure 1:**
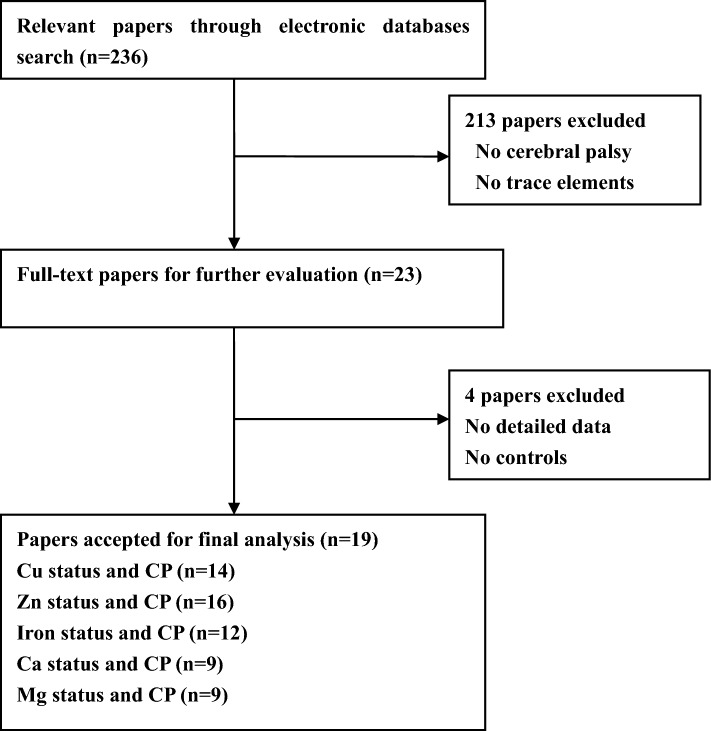
Flow diagram of study selection.

We firstly extracted 236 papers from the PubMed, Embase and Cochrane and Chinese Wan Fang databases. Most of these papers were removed due to that they were not associated with CP or Cu/Zn/Iron/Ca/Mg levels. After full-text review of the remaining studies, three studies were excluded due to the lack of detailed data. One study was excluded because it did not include control group. Finally, nineteen studies^8,13–30^ were included in this meta-analysis. A flow diagram showing the study selection is presented in Fig. 1.

### Study characteristics

The characteristics of the nineteen enrolled studies are shown in Table 1. They were published between 1989 and 2022. Fourteen studies were about Cu, sixteen for Zn, twelve for iron, nine for Ca, and nine for Mg. Seventeen studies adjusted for confounding factors. The participants were from Asians and Caucasians.

**Table 1:** Characteristics of studies enrolled in this meta-analysis.

Study	Study design	Ethnicity	Case/Control		Adjustment for confounding factors	Method of testing
			n	Trace element		
Cu						
Wang et al. 1989	CC	Asians	42/30	102.51 ± 25.46/121.28 ± 29.34 ug%	Age, gender	AAS
Liang et al. 1999 (urine)	CC	Asians	20/20	0.0578 ± 0.0162/0.0762 ± 0.016 ug/mL	Age, gender	AAS
Hao et al. 2000	CC	Asians	60/32	1.16 ± 0.25/23.14 ± 1.26 umol/L	Age, gender	ICP-AES
Li et al. 2003	CC	Asians	168/152	0.50 ± 0.27/0.71 ± 0.23 ug/mL	Age, gender	ICP-AES
Khalique et al. 2006	CC	Asians	95/93	6.224 ± 1.134/8.610 ± 1.760 ug/g	Age	sPectroPhotometer
Yuan et al. 2007	CC	Asians	128/128	14 ± 3.71/15 ± 3.26 mmol/L	Age, gender	AS
Hu et al. 2008	CC	Asians	131/130	3.60 ± 0.54/3.80 ± 0.51 ug/mL	Age, gender, Habits	AAS
Chen et al. 2010	CC	Asians	36/20	14.71 ± 3.79/22.06 ± 1.93 umol/L	Age, gender	AAS
Li et al. 2014	CC	Asians	53/60	1.071 ± 0.231/1.139 ± 0.372 ug/mL	Age, gender	AAS
Kalra et al. 2015	CC	Asians	50/50	106.18 ± 38.26/128.82 ± 20.23 ug/dl	Age, gender	Colorimetric method
Li et al. 2016	CC	Asians	217/219	18.38 ± 3.90/19.23 ± 4.07 umol/L	Age, gender	AS
Tang et al. 2016	CC	Asians	20/20	8.76 ± 1.48/20.31 ± 3.79 umol/L	Age, gender	–
Tinkov et al. 2021	CC	Caucasians	71/84	1.126 ± 0.193/1.195 ± 0.22 ug/ml	Age, gender	ICP-MS
Carman et al. 2022	CC	Caucasians	303/95	122.25 ± 29.21/125.18 ± 33.52 ug/dl	–	Colorimetric method
Zn						
Wang et al. 1989	CC	Asians	42/30	81.64 ± 12.47/96.19 ± 9.61 ug%	Age, gender	AAS
Liang et al. 1999 (urine)	CC	Asians	20/20	0.558 ± 0.22/0.775 ± 0.195 mg/L	Age, gender	AAS
Hao et al. 2000	CC	Asians	60/32	3.81 ± 0.96/24.43 ± 1.07 umol/L	Age, gender	ICP-AES
Li et al. 2003	CC	Asians	168/152	3.04 ± 1.05/5.83 ± 1.76 ug/mL	Age, gender	ICP-AES
Khalique et al. 2006	CC	Asians	95/93	143.9 ± 21.7/140.7 ± 27.7 ug/g	Age	sPectroPhotometer
Yuan et al. 2007	CC	Asians	128/128	78 ± 24/81 ± 29 mmol/L	Age, gender	AS
Hu et al. 2008	CC	Asians	131/130	2.80 ± 1.20/3.50 ± 1.40 ug/mL	Age, gender, habits	AAS
Yang et al. 2009	CC	Asians	277/311	3.5 ± 0.9/4.7 ± 1.3 umol/L	Age, gender	AAS
Chen et al. 2010	CC	Asians	36/20	67.96 ± 15.06/119.14 ± 29.70 umol/L	Age, gender	AAS
Li et al. 2014	CC	Asians	53/60	0.889 ± 0.295/1.229 ± 0.318 ug/mL	Age, gender	AAS
Kalra et al. 2015	CC	Asians	50/50	12.32 ± 4.95/13.30 ± 4.20 umol/l	Age, gender	Colorimetric method
Li et al. 2016	CC	Asians	200/340	47.19 ± 1.53/67.77 ± 1.60 umol/L	Age, gender	AAS
Li et al. 2016	CC	Asians	217/219	58.42 ± 14.47/61.6 ± 12.11 umol/L	Age, gender	AS
Tang et al. 2016	CC	Asians	20/20	9.57 ± 2.24/12.65 ± 2.56 umol/L	Age, gender	–
Tinkov et al. 2021	CC	Caucasians	71/84	0.928 ± 0.144/1.008 ± 0.164 ug/ml	Age, gender	ICP-MS
Carman et al. 2022	CC	Caucasians	303/95	123.69 ± 32.97/138.17 ± 34.58 ug/dl	–	Colorimetric method
Iron						
Li et al. 2003	CC	Asians	168/152	428 ± 43/451 ± 61 ug/mL	Age, gender	ICP-AES
Hao et al. 2004	CC	Asians	20/20	6.37 ± 2.03/15.57 ± 2.12 umol/L	Age, gender	TM
Khalique et al. 2006	CC	Asians	95/93	4.175 ± 2.018/6.882 ± 2.828 ug/g	Age	sPectroPhotometer
Yuan et al. 2007	CC	Asians	128/128	41 ± 7.29/53 ± 6.37 mmol/L	Age, gender	AS
Hu et al. 2008	CC	Asians	131/130	141.01 ± 54.94/161.48 ± 51.45 ug/mL	Age, gender, habits	AAS
Chen et al. 2010	CC	Asians	36/20	7.09 ± 0.87/8.15 ± 1.14 umol/L	Age, gender	AAS
Li et al. 2014	CC	Asians	53/60	0.757 ± 0.218/1.141 ± 0.307 ug/mL	Age, gender	AAS
Kalra et al. 2015	CC	Asians	50/50	12.60 ± 5.85/20.86 ± 3.29 umol/l	Age, gender	Colorimetric method
Li et al. 2016	CC	Asians	217/219	7.31 ± 1.29/7.55 ± 0.89 mmol/L	Age, gender	AS
Tang et al. 2016	CC	Asians	20/20	10.28 ± 2.56/20.75 ± 6.21 umol/L	Age, gender	–
Tinkov et al. 2021	CC	Caucasians	71/84	1.434 ± 0.662/1.399 ± 0.516 ug/ml	Age, gender	ICP-MS
Carman et al. 2022	CC	Caucasians	303/95	97.75 ± 74.17/100.77 ± 98.5 ug/dl	–	Colorimetric method
Ca						
Peng et al. 2005	CC	Asians	80/56	253.15 ± 68.84/288.44 ± 87.28 mmol/L	Age, gender	AAS
Khalique et al. 2006	CC	Asians	95/93	651.1 ± 135.6/521.2 ± 79.1 ug/g	Age	sPectroPhotometer
Yuan et al. 2007	CC	Asians	128/128	1.01 ± 0.22/1.29 ± 0.31 mmol/L	Age, Gender	AS
Hu et al. 2008	CC	Asians	131/130	18.21 ± 4.80/65.60 ± 22.11 ug/mL	Age, gender, Habits	AAS
Chen et al. 2010	CC	Asians	36/20	1.31 ± 0.127/1.83 ± 0.26 umol/L	Age, gender	AAS
Li et al. 2016	CC	Asians	217/219	1.81 ± 0.17/1.87 ± 0.23 mmol/L	Age, gender	AS
Tang et al. 2016	CC	Asians	20/20	1.48 ± 0.41/1.82 ± 0.51 mmol/L	Age, gender	–
Tinkov et al. 2021	CC	Caucasians	71/84	106 ± 9.2/106.2 ± 8 ug/ml	Age, gender	ICP-MS
Carman et al. 2022	CC	Caucasians	303/95	9.52 ± 0.84/9.42 ± 0.8 mg/dl	–	Colorimetric method
Mg						
Khalique et al. 2006	CC	Asians	90/93	216.2 ± 53/159.9 ± 49.10 ug/g	Age	sPectroPhotometer
Yuan et al. 2007	CC	Asians	128/128	1.01 ± 0.31/1.12 ± 0.40 mmol/L	Age, gender	AS
Hu et al. 2008	CC	Asians	131/130	49.35 ± 8.73/56.22 ± 6.43 ug/mL	Age, gender, habits	AAS
Chen et al. 2010	CC	Asians	36/20	1.47 ± 0.518/1.45 ± 0.08 umol/L	Age, gender	AAS
Schoendorfer et al. 2013	CC	Caucasians	15/24	0.65 ± 0.85/1.36 ± 1.24 z score	–	sPectroPhotometer
Kalra et al. 2015	CC	Asians	50/50	1.97 ± 0.41/2.19 ± 0.29 mg/dl	Age, gender	Colorimetric method
Li et al. 2016	CC	Asians	217/219	1.49 ± 0.23/1.51 ± 0.19 mmol/L	Age, gender	AS
Tinkov et al. 2021	CC	Caucasians	71/84	23.2 ± 2/23 ± 1.9 ug/ml	Age, gender	ICP-MS
Carman et al. 2022	CC	Caucasians	303/95	2.11 ± 0.27/2.12 ± 0.26 mmol/l	–	Colorimetric method

*CC* case–control, *Cu* copper, *Zn* zinc, *Ca* calcium, *Mg* magnesium, *ICP-AES* inductively coupled plasma atomic emission spectrometry, *AAS* atomic absorption spectroscopy, *AS* Anodic stripping, *TM* turbidimetric method.

### Cu level in CP and controls

**Figure 2:**
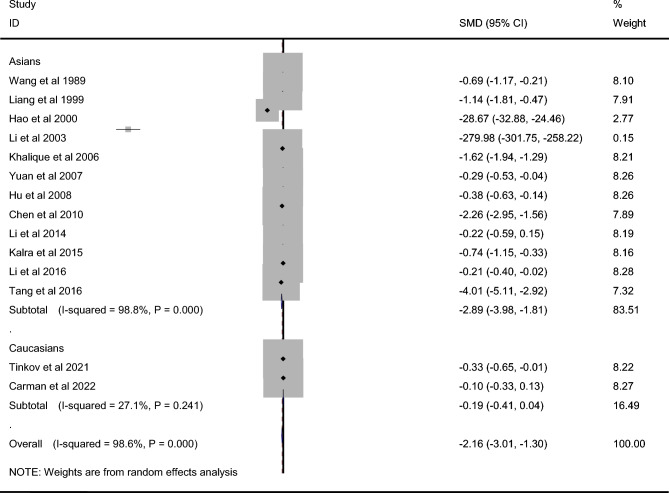
Difference of Cu level between CP and control.

A total of 1394 CP cases and 1133 controls were included for testing the Cu level. Atomic absorption spectroscopy (AAS) was used in testing the Ca level in five studies with anodic stripping (AS) in two studies, and inductively coupled plasma atomic emission spectrometry (ICP-AES) in two studies. CP cases showed markedly lower Cu level than that in controls among overall populations (SMD =  − 2.156, 95% CI − 3.013 to − 1.299, *P* < 10^−4^, Table 2, Fig. 2) and Asians (SMD =  − 2.893, 95% CI − 3.977 to − 1.809, *P* < 10^−4^, Table 2, Fig. 2). No significant difference of Cu status between CP and controls among Caucasians (SMD =  − 0.188, 95% CI − 0.412 to 0.037, *P* = 0.101, Table 2, Fig. 2) was observed. Sensitivity analysis did not change the overall results significantly (95% CI − 3.610 to − 0.531). Publication bias was observed (*P* < 10^−4^, funnel plot in Supplemental Material 1). Trim and fill analysis showed that addition of 4 virtual studies still yielded significant heterogeneity without changing overall result markedly.

**Table 2:** Association between the status of trace elements and cerebral palsy.

Trace elements	Studies	Q test P-value	Model selected	SMD (95% CI)	P-value
Cu					
Overall	14	< 10^−4^	Random	− 2.156 (− 3.013 to − 1.299)	< 10^−4^
Asians	12	< 10^−4^	Random	− 2.893 (− 3.977 to − 1.809)	< 10^−4^
Caucasians	2	0.241	Fixed	− 0.188 (− 0.412 to 0.037)	0.101
Zn					
Overall	16	< 10^−4^	Random	− 2.223 (− 2.966 to − 1.480)	< 10^−4^
Asians	14	< 10^−4^	Random	− 2.559 (− 3.436 to − 1.683)	< 10^−4^
Caucasians	2	0.687	Fixed	− 0.462 (− 0.650 to − 0.274)	< 10^−4^
Iron					
Overall	12	< 10^−4^	Random	− 1.092 (− 1.513 to − 0.672)	< 10^−4^
Asians	10	< 10^−4^	Random	− 1.336 (− 1.807 to − 0.865)	< 10^−4^
Caucasians	2	0.627	Fixed	− 0.004 (− 0.190 to 0.182)	0.968
Ca					
Overall	9	< 10^−4^	Random	− 0.757 (− 1.475 to − 0.040)	0.038
Asians	7	< 10^−4^	Random	− 1.000 (− 1.950 to − 0.051)	0.039
Caucasians	2	0.471	Fixed	0.070 (− 0.116 to 0.257)	0.459
Mg					
Overall	9	< 10^−4^	Random	− 0.139 (− 0.504 to 0.226)	0.455
Asians	6	< 10^−4^	Random	− 0.131 (− 0.663 to 0.401)	0.629
Caucasians	3	0.139	Fixed	− 0.074 (− 0.361 to 0.213)	0.614

*Cu* copper, *Zn* zinc, *Ca* calcium, *Mg* magnesium, *SMD* standard mean difference.

### Zn level in CP and controls

**Figure 3:**
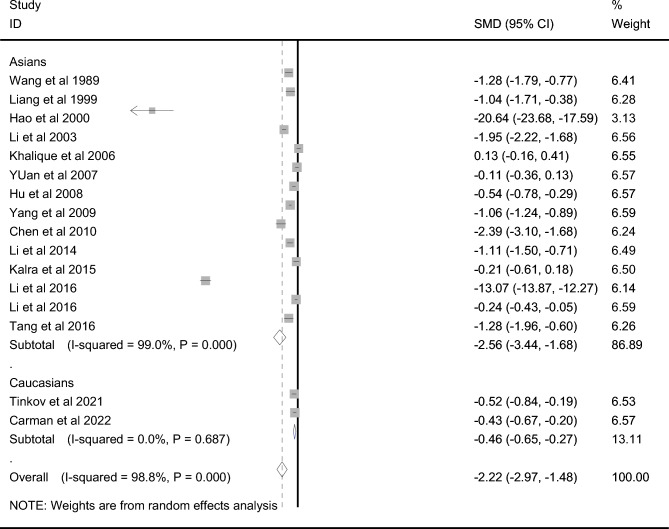
Difference of Zn level between CP and control.

A total of 1871 CP cases and 1784 controls were included for testing the Zn level. AAS was used in testing the Zn level in seven studies with AS in two studies, and ICP-AES in two studies. CP cases showed markedly lower Zn level than that in controls among overall populations (SMD =  − 2.223, 95% CI − 2.966 to − 1.480, *P* < 10^−4^, Fig. 3), Asians (SMD =  − 2.559, 95% CI − 3.436 to − 1.683, *P* < 10^−4^, Fig. 3), and Caucasians (SMD =  − 0.462, 95% CI − 0.650 to − 0.274, *P* < 10^−4^, Fig. 3). Sensitivity analysis did not change the overall results significantly (95% CI − 3.240 to − 0.717). Publication bias was observed (*P* = 0.023, funnel plot in Supplemental Material 2). Trim and fill analysis showed that addition of 5 virtual studies still yielded significant heterogeneity without changing overall result markedly.

### Iron level in CP and controls

**Figure 4:**
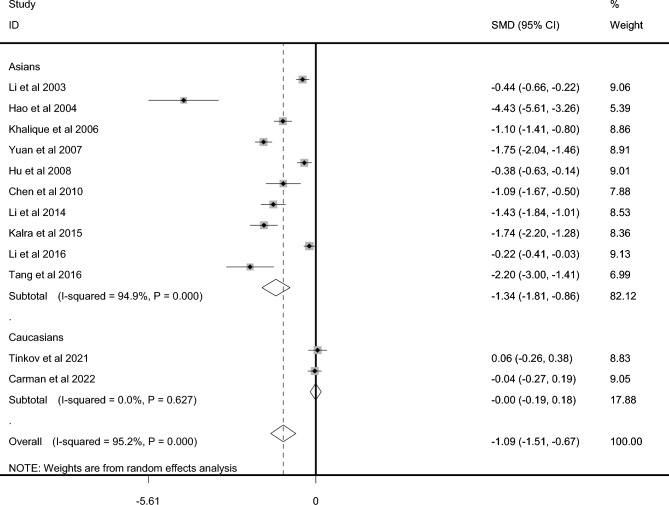
Difference of iron level between CP and control.

A total of 1292 CP cases and 1071 controls were included for testing the iron level. AAS was used in testing the iron level in three studies with AS in two studies, ICP-AES in one study, turbidimetric method (TM) in one study. CP cases showed markedly lower iron level than that in controls among overall populations (SMD =  − 1.092, 95% CI − 1.513 to − 0.672, *P* < 10 − ^4^, Fig. 4) and Asians (SMD =  − 1.336, 95% CI − 1.807 to − 0.865, *P* < 10^−4^, Fig. 4). No significant difference of Iron status between CP and controls among Caucasians (SMD =  − 0.004, 95% CI − 0.190 to 0.182, *P* = 0.968, Table 2, Fig. 4) was observed. Sensitivity analysis did not change the overall results significantly (95% CI − 1.666 to − 0.500). Publication bias was observed (*P* = 0.023, funnel plot in Supplemental Material 3). Trim and fill analysis showed that it did not need addition of virtual studies.

### Ca level in CP and controls

**Figure 5:**
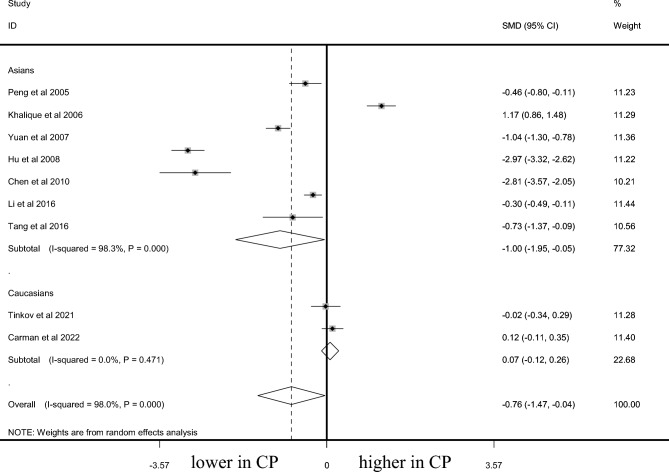
Difference of Ca level between CP and control.

A total of 1081 CP cases and 845 controls were included for testing the Ca level. AAS was used in testing the Ca level in three studies with AS in two studies. CP cases demonstrated significantly lower Ca level than that in controls among overall populations (SMD =  − 0.757, 95% CI − 1.475 to − 0.040, *P* = 0.038, Fig. 5) and Asians (SMD =  − 1.000, 95% CI − 1.950 to − 0.051, *P* = 0.039, Fig. 5). No significant difference of Ca status between CP and controls among Caucasians (SMD = 0.070, 95% CI − 0.116 to 0.257, *P* = 0.459, Table 2, Fig. 5) was observed. Sensitivity analysis changed the overall results a little (95% CI − 1.713 to 0.204). Publication bias was not observed (*P* = 0.346).

### Mg level in CP and controls

**Figure 6:**
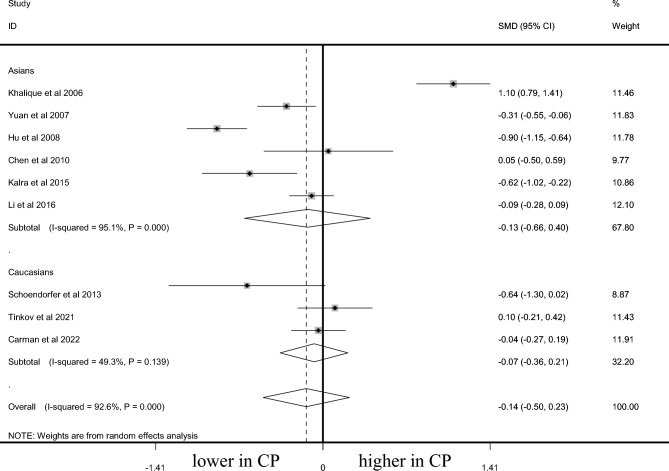
Difference of Mg level between CP and control.

A total of 1041 CP cases and 843 controls were included for testing the Mg level. AAS was used in testing the Mg level in two studies with AS in another two. No marked difference of Mg level between CP cases and controls was noted among overall populations (SMD =  − 0.139, 95% CI − 0.504 to 0.226, *P* = 0.455, Fig. 6), Asians (SMD =  − 0.131, 95% CI − 0.663 to 0.401, *P* = 0.629, Fig. 6), and Caucasians (SMD =  − 0.074, 95% CI − 0.361 to 0.213, *P* = 0.614, Fig. 6). Sensitivity analysis did not change the overall results (95% CI − 0.595 to 0.310). Publication bias was not observed (*P* = 0.984).

## Discussion

CP, one of the most common developmental disabilities during the childhood throughout the lifespan, is a clinical syndrome characterized by a motor disorder. CP has attracted much attention of doctors and parents of patients due to its harms to neurological and motor systems in children. Identification of the potential risk factors for CP susceptibility is helpful for the early prevention and treatment of CP. Adequate micronutrient supply in early postnatal period may be an important tool for neuroprotection. Cu, Iron, Zn are shown to play significant role in proper neurodevelopment and brain functioning. Our meta-analysis showed that CP cases demonstrated significantly lower levels of Cu, Zn, iron and Ca than those in controls among overall populations and Asians, which indicated that the deficiency of Cu/Zn/iron/Ca should be paid more attention in the population with higher susceptibility to CP. The homeostasis of Cu/Zn/iron/Ca may be very important for neuroprotection. Early monitoring and intervention may be helpful for CP prevention and treatment.

Several facts may account for our findings. CP is a neurological disorder usually induced by preterm birth or infection. Metal ions are closely associated with the normal functioning of human body^31^. Trace elements deficiency is likely to cause the immune dysfunction, resulting in the increased risk of infection. Cu is a key cofactor for various enzymes, such as Cu/Zn superoxide dismutase, which plays an important role for neurological development^32^. Cu is also involved in the redox reactions^33^. CP cases are prone to Cu deficiency^34^. Suboptimal Cu status was shown to be associated with poor motor performance^35^. Cu deficiency is also known to be associated with higher susceptibility to traumatic brain^36^. Cu deficiency also affects the role of other cellular constituents involved in antioxidant activities, such as iron, selenium, and also plays an important role in diseases in which oxidative stress is elevated. Oxidative stress was involved in the brain injury. Hence, the disorder of Cu may cause brain dysfunction. For example, higher level of Cu was associated with decreased risk of Parkinson’s disease, which is the second most common neurodegenerative disease^37^.

Zn is necessary for the survival of various types of cells. Lots of enzymes exert the effects by creating bonds with Zn ions^38^. Zn plays a role in cell proliferation as an element of transcription factors and enzymes of DNA replication, Zn deficiency leads to a decline of Th1 immunity and promotes inflammatory reactions. Zn is also present throughout the central nervous system, playing a role in synaptic transmission, neuroregulation and neuroprotection^39^. Zn also promotes spinal cord injury recovery through upregulating Zn transporter-1 and brain-derived neurotrophic factors^40^. Zn inhibits free radical by promoting metallothionein production. Meanwhile, Zn is crucial for retinol-binding protein synthesis and vitamin A mobilization. Zn plays a role in removing the heavy metals from the body, such as Pb, As and Hg, which are implicated in the pathophysiology of Parkinson’s disease^41^. Based on the comprehensive role of Zn in the body, Zn disorder may result in the unpredictable injuries including neurological lesions.

Iron is an important constituent of hemoglobin, which transfers oxygen. Iron deficiency leads to anemia. Thus, iron regulates and influences the activity of various organs, as well as the whole organism. Iron also exerts effects in the catalysis of enzymatic reactions^42^. Th cells maturation was impaired in children with iron-deficiency anemia and was regenerated by the supplementation of iron. Iron participates in the neurodevelopment^43^. Iron deficiency lowers the chances of recovery of the central nervous system and influences the children’s adaptation ability. Hence, iron homeostasis is important for neuroprotection.

Ca, an imPortant constituent of bones, Plays a vital role in the muscle contraction and relaxation, and also regulates the electrical conduction system of the heart^44^. Ca also regulates the function of enzymes and is associated with the metabolism of other trace elements^45^. Intracellular calcium concentration is an important regulator of several signaling mechanisms, which regulate various kinds of biological processes^46^. Alterations in calcium concentration play a vital role in muscle contraction and relaxation^47^. Dysregulated calcium levels have been observed in several muscular dystrophies, including Duchenne muscular dystrophies^48^. Metabolic bone disease is characterized by impaired Ca and P balance^49^. These previous evidence shows that Ca is closely associated with motor disorder and is a potential therapeutic target for CP.

Mg, another important constituent of bones, is an antagonist of Ca, prevents excessive acetylcholine release and stimulation at the neuromuscular junction. Notably, we observed null difference of Mg status between CP and controls, which may be due to that Mg was not directly associated with the neurodevelopment. Notably, Mg sulfate was commonly applied in obstetrics due to its prevention effect of eclamptic seizures^50^, which may also affect the Mg status in neonates. Further larger numbers of studies are needed to validate our findings.

Our findings supported the idea that nutritional status influences the neurodevelopment, neurocognitive performances, and later life health outcomes. Appropriate nutritional diet is important for lowering the adverse health consequences. Also, compared with the included previously published single studies, our study was a pooled investigation with robust significances. Although the positive association between Cu/Zn/iron/Ca and CP provided novel insight for CP prevention and therapy, several limitations should be considered. First, the between-study heterogeneity may distort the final results, the random-effects model decreased the influence of the heterogeneity. On the other hand, the sensitivity analysis did not change the overall results, which indicated that our conclusion was comparatively more robust. The participants were largely from Asians, which may limit the generalization of our results. More studies from different ethnicities may be recruited in the future for more robust results. Second, the publication bias was noted for the association between Cu/Zn/Iron and CP, trim and fill analysis did not change the overall results, indicating our results were comparatively solid. Finally, despite the significant differences of Cu/Zn/iron/Ca between CP and controls, the cause-effect relationship between trace elements levels and CP risk remains inconclusive. The enrolled participants were all children with a lower age, we speculated that trace elements deficiency may precede the CP onset. Due to the lack of specific age in our investigation, further larger number studies should be performed to make a meta-regression analysis regarding the age. Further exploring the influence of trace elements on CP occurrence will have greater clinical value. In terms of our findings, the following issues should be addressed: (1) time-series analysis of the alteration of trace elements in CP, (2) longitudinal observation of the association between trace elements levels and CP Progress, (3) clarification of the cause-effect relationship between trace elements status and CP risk in prospective studies.

In conclusion, our investigation indicates that CP cases demonstrated significantly lower levels of Cu/Zn/iron/Ca than those in healthy controls. Monitoring and intervention of Cu/Zn/iron/Ca disorder may be helpful for CP Prevention and therapy.

### Supplementary Information

Supplementary Information.

## Data Availability

The original extracted data can be obtained from the PubMed (https://pubmed.ncbi.nlm.nih.gov/), Embase (https://www.embase.com/) and Cochrane (https://www.cochranelibrary.com/) and Chinese Wan Fang databases (https://www.wanfangdata.com.cn/) (through searching the include article title for the full-text paper). The human data or humans were not directly involved in the study.

## References

[1] Spittle AJ, Morgan C, Olsen JE, Novak I, Cheong JLY (2018). Early diagnosis and treatment of cerebral palsy in children with a history of preterm birth. Clin. Perinatol..

[2] Hollung SJ, Bakken IJ, Vik T, Lydersen S, Wiik R, Aaberg KM, Andersen GL (2020). Comorbidities in cerebral palsy: A patient registry study. Dev. Med. Child Neurol..

[3] Sun L, Xia L, Wang M, Zhu D, Wang Y, Bi D, Song J, Ma C, Gao C, Zhang X, Sun Y, Wang X, Zhu C, Xing Q (2019). Variants of the OLIG2 Gene are associated with cerebral palsy in Chinese Han infants with hypoxic-ischemic encephalopathy. Neuromol. Med..

[4] Elmadfa I, Meyer AL (2019). The role of the status of selected micronutrients in shaping the immune function. Endocr. Metab Immune Disord. Drug Targets.

[5] Chatelain M, GasParini J, Haussy C, Frantz A (2016). Trace metals affect early maternal transfer of immune components in the feral pigeon. Physiol. Biochem. Zool..

[6] Wendołowicz A, Stefańska E, Ostrowska L (2018). Influence of selected dietary components on the functioning of the human nervous system. Rocz Panstw Zakl Hig..

[7] Zheng J, Jiang R, Chen M, Maimaitiming Z, Wang J, Anderson GJ, VulPe CD, Dunaief JL, Chen H (2018). Multi-copper ferroxidase-deficient mice have increased brain iron concentrations and learning and memory deficits. J. Nutr..

[8] Schoendorfer NC, Vitetta L, Sharp N, DiGeronimo M, Wilson G, Coombes JS, Boyd R, Davies PS (2013). Micronutrient, antioxidant, and oxidative stress status in children with severe cerebral palsy. JPEN.

[9] da Silva DCG (2022). Malnutrition and nutritional deficiencies in children with cerebral palsy: A systematic review and meta-analysis. Public Health.

[10] Ingran L, Nicola JR (2018). Magnesium as a neuroprotective agent: A review of its use in the fetus, term infant with neonatal encephalopathy, and the adult stroke patient. Dev Neurosci..

[11] Hough JP, Boyd RN, Keating JL (2010). Systematic review of interventions for low bone mineral density in children with cerebral palsy. Pediatrics..

[12] Moher D, Liberati A, Tezlaff J, Altman DG (2010). Preferred reporting items for systematic reviews and meta-analyses: The PRISMA statement. Int. J. Surg..

[13] Li Z (2003). Study on serum trace elements levels in 168 cerebral palsy cases. Chin. J. Misdiagn..

[14] Yuan H, Zhang G, Long Y, Chen D, Tao Y (2007). Study on elements (Ca, Zn, Fe, Cu, Mg) of blood in 128 CP. GuangDong Weiliang Yuansu Kexue..

[15] Liang H, Du W, Zhang X, Xing Q, Wang J, Jiang Y (1999). Study on urine zinc and copper in children with cerebral palsy. Trace Elem. Health Res..

[16] Chen X, Gao Z, Sun L, Dong X, Zhang T (2010). To research the microelement and immune function in children with cerebral palsy. Int. TCM Psychol. Syst. Bioinform..

[17] Wang H, Chen X, Li S, Wei C, Li Y, Li J (1989). Study on the change of serum zinc and copper levels in children with cerebral Palsy. Jia Mushi Med. Coll. Res..

[18] Hu Y (2008). Study on the serum trace elements in children with cerebral Palsy. Med. J. Chin. People’s Health.

[19] Li Q, Du C, Ren X (2016). Analysis of serum zinc level in children with cerebral Palsy. Chin. J. Pract. Nerv. Dis..

[20] Hao Q, Gao Y, Li F (2004). Study on the change of serum iron in children with cerebral Palsy. China Clin. Rehabil..

[21] Li M, Wu J, Zhang H, Yan J, Chen Q, Xiong C (2014). Nutritional status and intervention for children with cerebral palsy. Chin. J. Rehabil. Theory Pract..

[22] Li L (2016). Association between trace elements and growth/development in children with cerebral palsy. Chin. J. Pract. Nerv. Dis..

[23] Peng G (2005). Study on the calcium and bone density in children with cerebral palsy. Stud. Trace Elem. Health.

[24] Yang H, Li Q, Wang K, Wang J, Tang H, Wang L, Wang X (2009). Association between zinc and children with cerebral palsy. Chin. J. Rehabil. Theory Pract..

[25] Hao Q, Guo X, Wu T, Dong J (2000). Association between serum trace elements and cause of Children with cerebral palsy. Chin. J. Rehabil. Theory Pract..

[26] Tang Y (2016). Study on the change of serum trace elements and oxidative stress in children with severe cerebral palsy. Chin. J. Pract. Nerv. Dis..

[27] Tinkov AA, Skalnaya MG, Skalny AV (2021). Serum trace element and amino acid profile in children with cerebral palsy. J. Trace Elem. Med. Biol..

[28] Khalique A, Shah MH, Jaffar M, Shaheen N, Tariq SR, Manzoor S (2006). Multivariate analysis of the selected metals in the hair of cerebral palsy patients versus controls. Biol. Trace Elem. Res..

[29] Carman KB, Aydın K, Kilic Aydin B, Cansu A, Direk MC, Durmus S, Dündar NO, GencPinar P, Gungor S, Gurkas E, Hur O, Karadag M, Karademir CN, Ozkan Kart P, Okuyaz C, Oz NA, Peduk Y, Per H, Serin MH, Tekgul H, Unay B, Yarar C, Yildirim GK (2022). Evaluation of micronutrient levels in children with cerebral palsy. Pediatr. Int..

[30] Kalra S, Aggarwal A, Chillar N, Faridi MMA (2015). comparison of micronutrient levels in children with cerebral palsy and neurologically normal controls. Indian J. Pediatr..

[31] Polyudova TV, Eroshenko DV, Korobov VP (2018). Plasma, serum, albumin, and divalent metal ions inhibit the adhesion and the biofilm formation of Cutibacterium (propionibacterium) acnes. AIMS Microbiol..

[32] Lasiene J, Komine O, Fujimori-Tonou N, Powers B, Endo F, Watanabe S, Shijie J, Ravits J, Horner P, Misawa H, Yamanaka K (2016). Neuregulin 1 confers neuroprotection in SOD1-linked amyotrophic lateral sclerosis mice via restoration of C-boutons of spinal motor neurons. Acta NeuroPathol. Commun..

[33] Ognik K, Cholewińska E, Juśkiewicz J, Zduńczyk Z, Tutaj K, Szlązak R (2019). The effect of copper nanoparticles and copper (II) salt on redox reactions and epigenetic changes in a rat model. J. Anim. Physiol. Anim. Nutr..

[34] Janet Y (2010). Influence of copper on early development: Prenatal and postnatal considerations. Biofactors.

[35] Bumoko GMM, Sadiki NH, Rwatambuga A, Kayembe KP, Okitundu DL, Ngoyi DM, Muyembe JJT, Banea JP, Boivin MJ, Tshala-Katumbay D (2015). Lower serum levels of selenium, copper, and zinc are related to neuromotor impairments in children with konzo. J. Neurol. Sci..

[36] Klevay LM (2013). Myelin and traumatic brain injury: The copper deficiency hypothesis. Med. Med. Hypotheses.

[37] Kim MJ, Oh SB, Kim J, Kim K, Ryu HS, Kim MS, Ayton S, Bush AI, Lee JY, Chung SJ (2018). Association of metals with the risk and clinical characteristics of Parkinson's disease. Parkinsonism Relat. Disord..

[38] Khan MF, Kundu D, Hazra C, Patra S (2019). A strategic approach of enzyme engineering by attribute ranking and enzyme immobilization on zinc oxide nanoparticles to attain thermostability in mesophilic *Bacillus*
*subtilis* lipase for detergent formulation. Int. J. Biol. Macromol..

[39] Justice JA, Manjooran DT, Yeh CY, Hartnett-Scott KA, Schulien AJ, Kosobucki GJ, Mammen S, Palladino MJ, Aizenman E (2018). Molecular neuroprotection induced by zinc-dependent expression of hepatitis C-derived protein NS5A targeting Kv21 potassium channels. J. Pharmacol. Exp. Ther..

[40] Li X, Chen S, Mao L, Li D, Xu C, Tian H, Mei X (2019). Zinc improves functional recovery by regulating the secretion of granulocyte colony stimulating factor from microglia/ macrophages after spinal cord injury. Front. Mol. Neurosci..

[41] Tamegart L, Abbaoui A, Makbal R, Zroudi M, Bouizgarne B, Bouyatas MM, Gamrani H (2019). Crocus sativus restores dopaminergic and noradrenergic damages induced by lead in Meriones Shawi: A possible link with Parkinson's disease. Acta Histochem..

[42] Stoyanovsky DA, Tyurina YY, Shrivastava I, Bahar I, Tyurin VA, Protchenko O, Jadhav S, Bolevich SB, Kozlov AV, Vladimirov YA, Shvedova AA, PhilPott CC, Bayir H, Kagan VE (2019). Iron catalysis of lipid peroxidation in ferroptosis: Regulated enzymatic or random free radical reaction?. Free Radic. Biol. Med..

[43] Wang Y, Wu Y, Li T, Wang X, Zhu C (2019). Iron metabolism and brain development in premature infants. Front. Physiol..

[44] Nishikawa K, Dutta S, DuVall M, Nelson B, Gage MJ, Monroy JA (2020). Calcium-dependent titin- thin filament interactions in muscle: Observations and theory. J. Muscle Res. Cell Motil..

[45] Gaglianone RB, Bloise FF, Ortiga-Carvalho TM, Quirico-Santos T, Costa ML, Mermelstein C (2020). ComParative study of calcium and calcium-related enzymes with differentiation markers in different ages and muscle types in mdx mice. Histol. Histopathol..

[46] Pingel J, Vandenrijt J, KamPmann ML, Andersen JD (2022). Altered gene expression levels of genes related to muscle function in adults with cerebral palsy. Tissue Cell.

[47] Wahid M, Saqib F, Akhtar S, Ali A, Tallei TE, Simal-Gandara J (2023). Mechanistic insights of *Cucumis*
*melo* L. seeds for gastrointestinal muscle spasms through calcium signaling pathway-related gene regulation networks in WGCNA and in vitro, in vivo studies. Comput. Biol. Med..

[48] Marks AR (2023). Targeting ryanodine receptors to treat human diseases. J Clin Invest..

[49] Rayannavar A, Calabria AC (2020). Screening for metabolic bone disease of prematurity. Semin. Fetal Neonatal Med..

[50] Brookfield KF, Mbata O (2023). Magnesium sulfate use in pregnancy for preeclampsia prophylaxis and fetal neuroprotection: Regimens in high-income and low/middle-income countries. Obstet. Gynecol. Clin. North. Am..

