# Supplementary material for: Association between Cu/Zn/Iron/Ca/Mg levels and cerebral palsy: a pooled-analysis
Source: Sci Rep. 2023 Oct 27;13:18427. doi: 10.1038/s41598-023-45697-w (PMC10611728; doi:10.1038/s41598-023-45697-w)
Supplement: Supplementary file 1 — Supplementary Information. [file 41598_2023_45697_MOESM1_ESM.docx]

Funnel plot with pseudo 95% confidence limits

SMD

s.e. of: SMD

0

5

10

-300

-200

-100

0

**Supplemental material 1:** Funnel plot for the studies about Cu level in CP and controls

Funnel plot with pseudo 95% confidence limits

SMD

s.e. of: SMD

0

.5

1

1.5

-20

-10

0

**Supplemental material 2:** Funnel plot for the studies about Zn level in CP and controls

Funnel plot with pseudo 95% confidence limits

SMD

s.e. of: SMD

0

.2

.4

.6

-4

-2

0

2

**Supplemental material 3:** Funnel plot for the studies about Iron level in CP and controls
